# Genetic associations in ankylosing spondylitis: circulating proteins as drug targets and biomarkers

**DOI:** 10.3389/fimmu.2024.1394438

**Published:** 2024-05-21

**Authors:** Ye Zhang, Wei Liu, Junda Lai, Huiqiong Zeng

**Affiliations:** ^1^ Traditional Chinese Medicine Department of Immunology, Women & Children Health Institute Futian Shenzhen, Shenzhen, China; ^2^ First Teaching Hospital of Tianjin University of Traditional Chinese Medicine, National Clinical Research Center for Chinese Medicine Acupuncture and Moxibustion, Tianjin, China; ^3^ Department of Human Life Sciences, Beijing Sport University, Beijing, China

**Keywords:** Ankylosing spondylitis, mendelian randomization, drug target, biomarker, colocalization, Phenomewide Association Study, (PheWAS), proteomics

## Abstract

**Background:**

Ankylosing spondylitis (AS) is a complex condition with a significant genetic component. This study explored circulating proteins as potential genetic drug targets or biomarkers to prevent AS, addressing the need for innovative and safe treatments.

**Methods:**

We analyzed extensive data from protein quantitative trait loci (pQTLs) with up to 1,949 instrumental variables (IVs) and selected the top single-nucleotide polymorphism (SNP) associated with AS risk. Utilizing a two-sample Mendelian randomization (MR) approach, we assessed the causal relationships between identified proteins and AS risk. Colocalization analysis, functional enrichment, and construction of protein-protein interaction networks further supported these findings. We utilized phenome-wide MR (phenMR) analysis for broader validation and repurposing of drugs targeting these proteins. The Drug-Gene Interaction database (DGIdb) was employed to corroborate drug associations with potential therapeutic targets. Additionally, molecular docking (MD) techniques were applied to evaluate the interaction between target protein and four potential AS drugs identified from the DGIdb.

**Results:**

Our analysis identified 1,654 plasma proteins linked to AS, with 868 up-regulated and 786 down-regulated. 18 proteins (AGER, AIF1, ATF6B, C4A, CFB, CLIC1, COL11A2, ERAP1, HLA-DQA2, HSPA1L, IL23R, LILRB3, MAPK14, MICA, MICB, MPIG6B, TNXB, and VARS1) that show promise as therapeutic targets for AS or biomarkers, especially MAPK14, supported by evidence of colocalization. PhenMR analysis linked these proteins to AS and other diseases, while DGIdb analysis identified potential drugs related to MAPK14. MD analysis indicated strong binding affinities between MAPK14 and four potential AS drugs, suggesting effective target-drug interactions.

**Conclusion:**

This study underscores the utility of MR analysis in AS research for identifying biomarkers and therapeutic drug targets. The involvement of Th17 cell differentiation-related proteins in AS pathogenesis is particularly notable. Clinical validation and further investigation are essential for future applications.

## Introduction

Ankylosing spondylitis (AS) is a chronic inflammatory joint disease that exhibits significant regional and population-based variations in prevalence, influenced primarily by genetic predisposition, shaping both disease susceptibility and severity (1). It commonly appears during adolescence or early adulthood and is more prevalent in men. The disease is notably associated with genetic factors, including the presence of the HLA-B27 antigen, and its prevalence varies significantly across different regions and populations (2).

In recent years, pharmaceutical research has made substantial progress, introducing new therapeutic options alongside conventional treatments such as non-steroidal anti-inflammatory drugs (NSAIDs) and biologic disease-modifying antirheumatic drugs (bDMARDs) (3). Notably, FDA-approved medications like tumor necrosis factor inhibitors (TNFi) and interleukin-17 inhibitors (IL-17i) have significantly enhanced the quality of life for those with AS (4). Despite these advancements, the quest for more effective treatments continues, especially for patients who do not respond adequately to existing therapies.

Pharmaceuticals often target specific proteins, and genetic variations affecting protein expression provide opportunities for drug repurposing and the development of new treatments (5). This study focuses on the potential of proteomic technologies to identify new therapeutic targets. Proteins, due to their diverse functions, serve as excellent candidates for uncovering disease biomarkers and therapeutic targets (6). Advances in genetic and proteomic analysis, such as Mendelian Randomization (MR) and Genome-wide Association Studies (GWAS), have facilitated the use of large-scale data to establish causality between genetic variations and disease, simulating the effects of randomized clinical trials. This approach helps minimize the influence of confounding factors and enhances the prediction of individual responses to treatment, thus supporting personalized medicine (7, 8). Despite the potential, the genomic basis for new drug targets in AS has remained largely unexplored. MR analysis of the drug target gene is a statistical approach used to evaluate whether a particular gene is associated with the therapeutic effects of a drug (9). It offers advantages over traditional research methods, using genetic variations to simulate randomized clinical trials, thus providing stronger causal evidence, reducing the impact of confounding variables, supporting high-throughput screening, saving time and resources, and advancing personalized medicine (10). This aids to gain a deeper understanding of the mechanism of action of the drug and enhances the prediction of individual responses to the drug. To our knowledge, the genomic evidence for potential drug targets in AS remains unexplored.

We used a genetic instrument comprising 1,949 circulating plasma proteins from extensive proteomics research. We obtained AS data from publicly available datasets. Using MR and colocalization analysis within the proteomic realm, we investigated the causal links between circulatory proteins and AS, identifying potential therapeutic targets. We also evaluated protein-protein interactions (PPI) and suitability for drug development, prioritizing treatment targets and biomarkers. This research sheds light on the causal associations between proteins and AS, offering new insights and therapeutic possibilities. Our findings contribute to a deeper understanding of AS and provide a foundation for the development of targeted therapies, representing a significant advancement in the treatment and prevention of this debilitating disease.

## Materials and methods

### Screening instrumental variables and data sources

In this study, genetic variants associated with protein abundance are differentiated into cis-acting and trans-acting types. The quantitative trait loci that act on CS (pQTLs) were typically located near the genes that encode the corresponding proteins (within 1Mb in this work) and have direct and independent biological effects (11). Proteins are more likely than other molecular traits to be used as drug targets, and MR analysis combined with pQTL as instrumental variables (IVs) is valuable for drug development in human genetics. We integrated the cis-pQTL data derived from prior studies conducted by Sun BB et al. (12–17) as our genetic IVs source. Significance thresholds were set at P< 5e-8 (with a minor allele frequency > 0.01) and a linkage disequilibrium (LD) threshold of *R* ^2^< 0.8, kb = 10,000, utilizing the two-sample MR package (version *0.5.7*) *R* (18). The explanatory power of IVs concerning exposure was quantified using the *F*-statistic as shown below:


$$F=\frac{R^{2}\times \left(N-1-k\right)}{\left(1-R^{2}\right)\times k}$$

$$R^{2}=2\times \text{MAF}\times \left(1-\text{MAF}\right)\times {\left(\frac{\text{β}}{\text{sd}}\right)}^{2}$$

**Figure 1:**
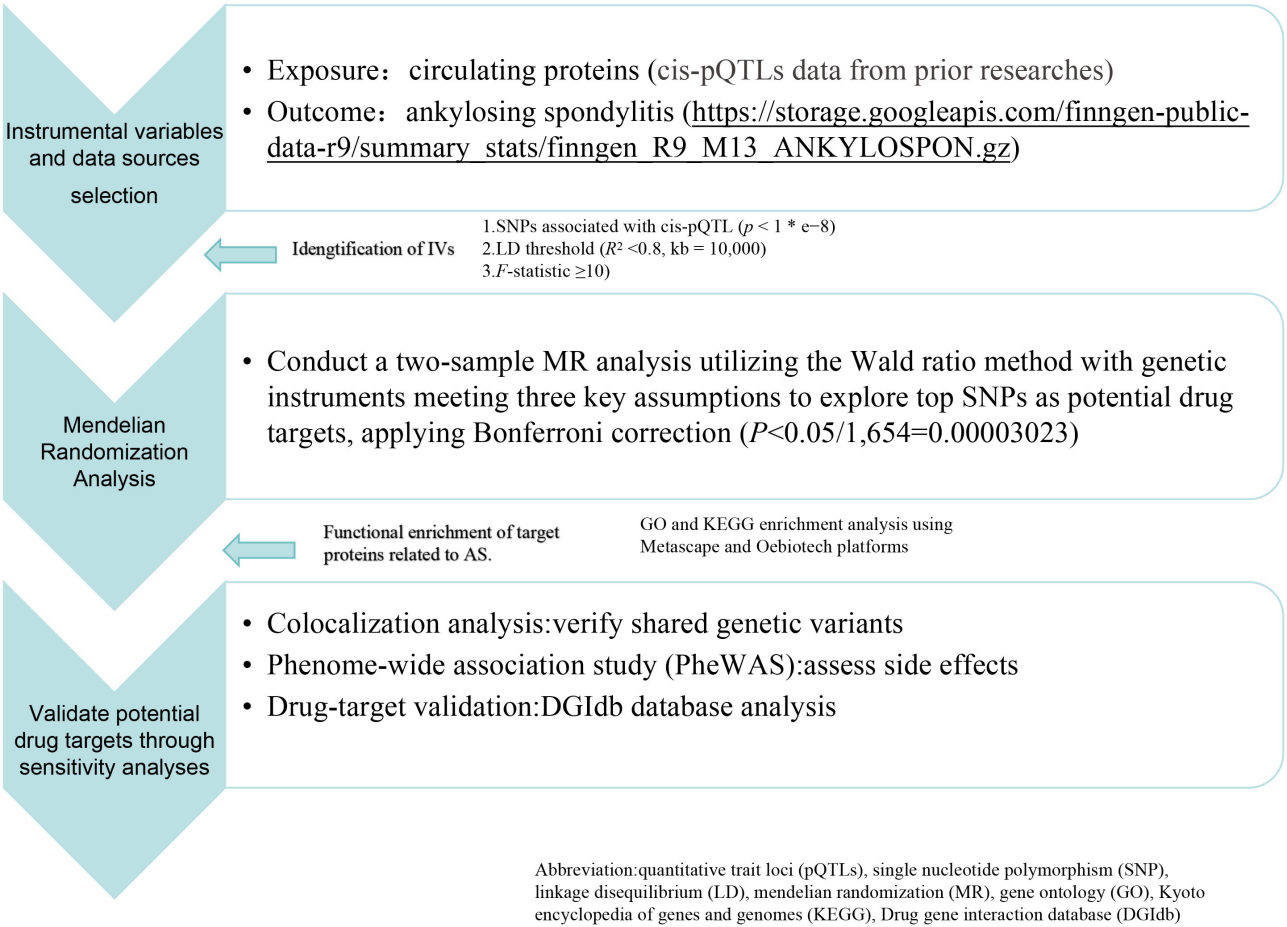
Flowchart of the Mendelian Randomization (MR) analysis framework.

In this formula, MAF represents the minimum allele frequency; β represents the effect size in GWAS for proteins and sd is its standard deviation (sd = se × $\sqrt{N}$ ); N is the total number of samples in the proteins GWAS; *k* is the number of IVs used (19). An *F*-statistic below 10 suggests a potentially weak IV strength, indicative of inadequate suitability for robust causal inference. Blood pQTLs associated with AS were identified from the latest public GWAS database as outcomes (https://storage.googleapis.com/finngen-public-data-r9/summary_stats/finngen_R9_M13_ANKYLOSPON.gz). We employed blood proteomic profiling data as SNPs to perform a two-sample MR analysis across two independent cohorts consisting of 2,860 AS cases and 270,964 controls. This methodological approach facilitates the identification of SNPs linked to specific protein expressions, advancing research into genetic variations associated with diseases. The workflow of the study is depicted in **Figure 1**.

### MR and statistical analysis

MR analysis mandates the fulfillment of three crucial assumptions: genetic instruments are strongly correlated with exposure, genetic instruments are independent of confounding factors, and genetic instruments solely influence the outcome through their impact on exposure. The Wald ratio (WR) method was employed for preliminary explorations of the top SNPs. (most significant SNPs) (20, 21). Where only a single IV was available with a *P*-value of<1e-05, the WR was computed. A Bonferroni correction was applied to the *P*-value (0.05/1,654 = 0.00003023) to mitigate the false discovery rate (FDR), and findings were articulated using statistical metrics (Odds Ratio and 95% Confidence Interval) (22). Throughout these analyses, only results with a statistically significant level (*P*< 0.05) were retained to ensure the reliability of the results.

### Colocalization analysis

Colocalization analysis serves as a methodological approach for investigating protein-protein interactions and functions (23). When comparing the subcellular localization patterns of proteins, we reveal their interrelationships. This analysis facilitates the assessment of whether disease-associated and genetic regulation of protein levels are driven by identical genetic variants. In our study, we used Bayesian colocalization analysis with default parameters from the “coloc” software package to assess the likelihood of two features sharing the same causal variation (24). We evaluated five hypotheses’ posterior probabilities (PPH) to ascertain whether two features are influenced by a common variant. The coloc.abf algorithm was applied, classifying proteins as first-tier (PPH4 > 0.8) with strong evidence of colocalization, second-tier (0.5< PPH4< 0.8) with moderate support, and others as third-tier targets based on prior research (25).

### Protein–protein interaction network and GO/KEGG analysis

The exploration of protein-protein interactions (PPI) was facilitated using the String database (https://string-db.org/) (26). It contains known protein interaction data from various sources, including experimental evidence and computational predictions. This step involved data preparation, access to the String database, extraction of the PPI network, and subsequent visualization and analytical interpretation of these interactions. The parameters for this study were configured as follows: the network edges were specifically interpreted as indicators of interaction evidence, while the active interaction sources included a range of methodologies such as textmining, experimental assays, Database entries, co-expression patterns, proximity in genomic context, Gene Fusion events, and co-occurrence statistics. The threshold for inclusion in the network was set at a medium confidence score of 0.400 to ensure relevance and reliability of the interactions.

Regarding the identification of candidate genes implicated in AS pathogenesis, genes derived from the Bonferroni correction statistical approach were pinpointed as key targets. Further insights into the biological pathways and functional attributions of these genes were gleaned through Gene Ontology (GO) and Kyoto Encyclopedia of Genes and Genomes (KEGG) enrichment analyses. These analyses were performed using Metascape (http://metascape.org) and the Oebiotech platform (https://cloud.oebiotech.cn/, last accessed on 12 January 2024) (27). These tools facilitated the visualization and interpretation of potential pathways involved in the disease’s progression and pathogenesis, enhancing our understanding of the molecular mechanisms at play (27, 28).

### Phenome-wide MR analysis

Phenome-wide mendelian randomization (PhenMR), integrating principles from both Mendelian Randomization (MR) and Phenome-Wide Association Studies (PheWAS), is employed to elucidate the causal relationships between genes and a spectrum of phenotypic traits (29). This method enables a broad assessment of genetic impacts across health and disease phenotypes, revealing gene-phenotype associations and facilitating the exploration of genetic functions and biological underpinnings.

To exclude potential pleiotropy of AS, we performed a PheWAS analysis using variants identified from the GWAS ATLAS and Pheno-Scanner databases (30–32). This analysis allows for the investigation of how the six primary proteins linked to AS are associated with various traits, shedding light on their roles in AS pathogenesis and their potential as therapeutic targets.

### Drugs investigation: DGIdb analysis

The drug gene interaction database (DGIdb) (https://dgidb.genome.wustl.edu) is a database that integrates drug-gene interaction data from 30 existing databases, helping researchers to explore existing data to generate hypotheses about how genes can be used in therapy or drug development (33). It allows users to search for drugs that target specific genes of interest, including FDA-approved, experimental, and investigational drugs, along with information on their indications, target genes, and interaction types. Our search within the DGIdb was guided by prior research, focusing on genes associated with potential treatment modalities for AS (34). Ethical approval had been obtained from their institutional review board in each study, and all participants had provided informed consent, obviating the need for additional ethical approval.

### Molecular docking analysis of MAPK14 with selected pharmacological agents

The three-dimensional configuration of the MAPK14 protein was sourced from the Protein Data Bank (https://www1.rcsb.org, PDB ID: 6zwp) and refined by removing non-essential entities using PyMOL software, version *2.5.4* (35, 36). The molecular structures of four therapeutic agents, as identified in the Drug Gene Interaction Database (DGIdb), along with a co-crystallized ligand, were retrieved from the PubChem repository, (https://pubchem.ncbi.nlm.nih.gov) and subjected to structural optimization employing OpenBabel (version *3.1.1*) and ORCA (version 5.03) (37–39). The root mean square deviation (RMSD) values for co-crystallized ligand structures were calculated before and after docking using PyMOL software. An RMSD value of less than 2 Å indicates successful methodological validation. Computational assessments were conducted using the B3LYP hybrid functional combined with the def2-TZVP basis set. To prepare for molecular docking, modifications to enhance hydrogen bonding and molecular flexibility were executed using Auto Dock Tools version *1.5.6*. The docking procedure itself was facilitated by Autodock Vina software (version *1.2.0*), employing the Lamarckian genetic algorithm to optimize interaction predictions (40). Specific parameters for the docking grid were set with a central point at coordinates (X, Y, Z) = (6, 0, 19) and dimensions spanning (X×Y×Z) = (26×26×24), with an exhaustiveness setting of 25 to ensure thorough sampling.

## Results

### Identifying plasma proteins related to AS

**Figure 2:**
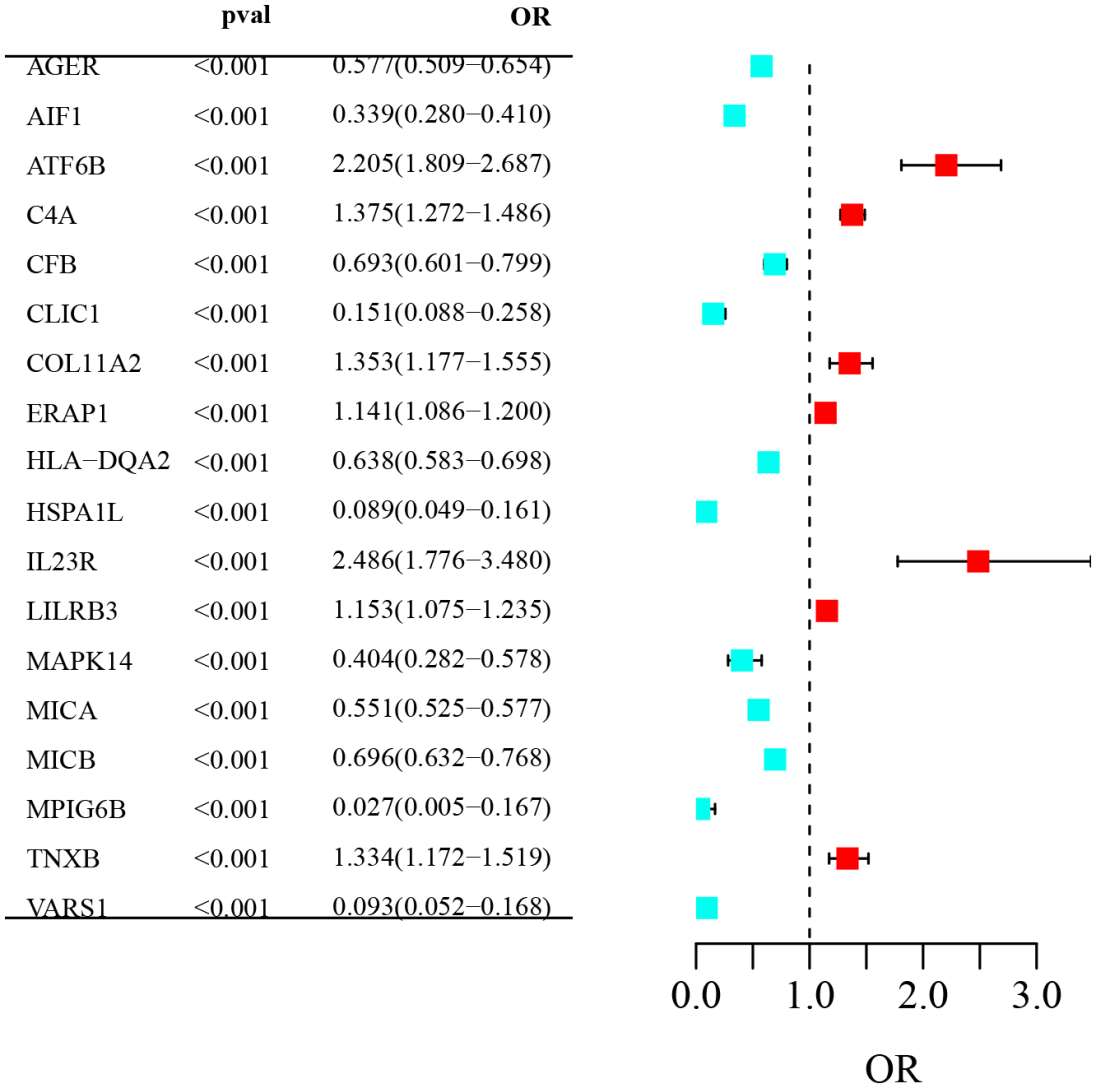
Forest plot of odds ratios (OR) for genetic variants associated with AS: The forest plot visualizes OR and 95% confidence intervals for genes associated with AS. Listed genes show significant ORs (*P*< 0.001). Red indicates increased AS risk; green indicates reduced risk. The vertical dashed line denotes OR=1, the threshold for risk significance.

This study explored how certain proteins, for which genetic markers are known, are related to the risk of AS. We focused on 1,949 proteins that have identifiable genetic signals known as pQTLs and examined their influence on the development and severity of AS. **Figure 2** provides a summary of these findings. We match a single cis-SNP for each protein by integrating genetic data from pQTL, and we identify 1,654 unique circulating plasma proteins that met the criteria for having a causal relationship with AS (**Supplementary Table S1**). Among these, 868 proteins showed a positive causal relationship with AS, while 786 proteins showed a negative causal relationship with AS. This suggested that specific genetic variations may be associated with the risk of AS by influencing the expression or function of these plasma proteins, thus affecting the occurrence of AS.

### Exploring causal plasma proteins on AS

To ensure the reliability of our findings, we used the Bonferroni correction method to adjust for multiple comparisons, setting a strict significance threshold (P value = 0.05/1,654, *P<* 3.03E-05). Among the 1,654 plasma proteins mentioned above, we identified 18 that were significantly associated with the risk of developing AS. These proteins included AGER, AIF1, ATF6B, C4A, CFB, CLIC1, COL11A2, ERAP1, HLA-DQA2, HSPA1L, IL23R, LILRB3, MAPK14, MICA, MICB, MPIG6B, TNXB and VARS1. Specifically, seven of these proteins were associated with an increased risk of AS, including ATF6B, C4A, COL11A2, etc. Additionally, 11 proteins were found to lower the risk of AS, such as AIF1, MAPK14, MICA, MICB, etc. Interestingly, the most significant factors influencing AS were the proteins IL23R (OR: 2.49), ATF6B (OR: 2.21), and C4A (OR: 1.38). These findings provide compelling information for further research on the role of these proteins in AS, warranting further investigation (**Table 1**).

**Table 1:** Causal plasma proteins in ankylosing spondylitis (AS).

id.exposure	b	se	*P*-val	or	or_lci95	or_uci95
IL23R	0.910738095	0.17152	1.10E-07	2.486156877	1.776346799	3.479599828
ATF6B	0.790523956	0.100935112	4.80E-15	2.204551213	1.808849735	2.686815801
C4A	0.318389759	0.039770145	1.19E-15	1.374912042	1.271808972	1.486373476
COL11A2	0.302283909	0.070992156	2.06E-05	1.352945285	1.177200641	1.554926901
TNXB	0.288305453	0.066304125	1.37E-05	1.334164767	1.171575435	1.519317981
LILRB3	0.142045886	0.035405812	6.02E-05	1.152629536	1.075354628	1.235457414
ERAP1	0.132335882	0.025460529	2.02E-07	1.141491661	1.085926181	1.199900357
MICB	-0.361808092	0.049643729	3.14E-13	0.696416003	0.631845864	0.76758475
CFB	-0.36645838	0.072386049	4.14E-07	0.693184987	0.601496215	0.79885029
HLA-DQA2	-0.450084251	0.046000788	1.32E-22	0.637574433	0.582605033	0.697730254
AGER	-0.549864917	0.063904962	7.67E-18	0.577027752	0.509096017	0.654024026
MICA	-0.596719252	0.024205555	3.49E-134	0.550615106	0.525102321	0.577367463
MAPK14	-0.906780645	0.183013878	7.24E-07	0.40382218	0.28210164	0.578062408
AIF1	-1.081463415	0.097452654	1.29E-28	0.33909892	0.280138651	0.410468449
CLIC1	-1.893118036	0.274137029	4.99E-12	0.150601495	0.087999283	0.257738581
VARS1	-2.370585134	0.297909331	1.76E-15	0.093426043	0.052105362	0.167514922
HSPA1L	-2.424102426	0.304649057	1.76E-15	0.08855757	0.048741982	0.160897096
MPIG6B	-3.59552822	0.92036903	9.36E-05	0.027446182	0.004519059	0.166692427

Ankylosing spondylitis (AS), each identifier for the exposure factor being investigated, which in our study refers to specific plasma proteins (id.exposure), beta coefficient (b), standard error (se), P-value (P-val), lower confidence interval (lo_ci), upper confidence interval (up_ci), Odds Ratio (or), 95% confidence interval lower limit for odds ratio (or_lci95), 95% confidence interval upper limit for odds ratio (or_uci95).

### Enrichment and construction of gene networks of drug targets

**Figure 3:**
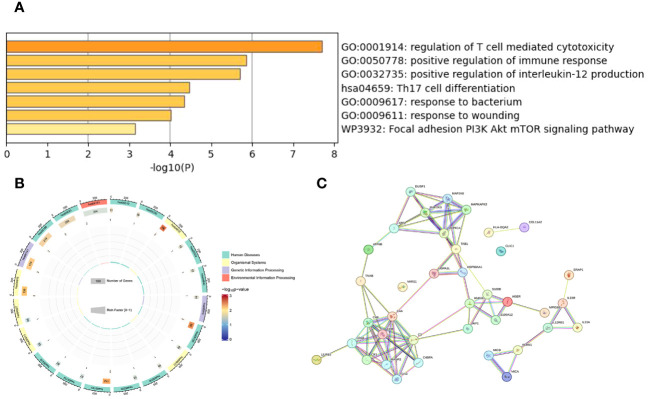
Enrichment and interaction analysis of AS-related genes: **(A)** The bar graph represents the -log10 transformed *P*-values of the enriched Gene ontology (GO) terms related to the biological processes in AS. It highlights significant processes such as immune response regulation and cytokine activity. **(B)** This circular plot represents the Kyoto Encyclopedia of Genes and Genomes (KEGG) pathway analysis, showing the pathways significantly enriched with genes related to AS. Each segment’s color gradient indicates the level of enrichment significance, with a richer color denoting a lower p-value and higher significance. The plot also quantifies the number of genes involved in each pathway. **(C)** Protein-protein interaction (PPI) network analysis conducted using the STRING database to reveal the interactions between proteins encoded by AS-related genes.

We conducted a Gene Ontology (GO) analysis on 18 shared protein targets, covering biological processes (BP), Molecular Function (MF) and Cellular Component (CC), as seen in **Figure 3A**. Notable GO terms included positive regulation of interleukin-12 production (GO:0032735), regulation of T cell-mediated cytotoxicity (GO:0001914). The KEGG enrichment analysis showed that Th17 cell differentiation signaling pathway was significantly enriched in target proteins (see **Figure 3B**). We also constructed a map showing interactions between these proteins, featuring 38 connection points and 82 connecting lines, which suggests a highly significant network based on a statistical measure (*P*-value:1.45e-12), as seen in **Figure 3C** using the String platform.

### Colocalization analysis

In our research, we performed a detailed analysis to study how causal genetic variations and proteins were connected to AS. This technique, known as co-localization analysis, helps us understand which genetic changes are likely to influence the disease and its treatment. Specifically, we found that four proteins—MAPK14, AIF1, ATF6B, and MICA— can serve as first-tier drug targets. HSPA1L and COL11A2 were second tiers, according to the results, while others were categorized as third-tier targets. Our findings suggested possible shared causal genetic variations between MAPK14 and AS (PPH4 = 0.977), while ATF6B could pose a risk factor for AS (PPH4 = 0.887). These results underscore our identification of several credible drug-related genes via MR and colocalization analysis, revealing a common genetic effect between target proteins and AS risk (**Table 2**).

**Table 2:** Results of the colocalization analysis.

Protein	N_SNPs	PPH0	PPH1	PPH2	PPH3	PPH4
AGER	3712	5.72E-127	0.0539114	7.44E-112	0.942793077	0.00329552
AIF1	3856	5.32E-96	0.00167447	6.92E-74	0.102649734	0.8956758
ATF6B	5359	0	2.76E-06	0	0.112731838	0.8872654
C4A	6478	1.23E-87	0.12798836	1.60E-35	0.867830945	0.004180694
CFB	4550	0	5.65E-32	0	1	9.20E-63
CLIC1	5123	4.51E-85	0.00515944	5.86E-58	0.994758254	8.23E-05
COL11A2	4456	0	6.27E-06	0	0.264638428	0.7353553
ERAP1	5769	1.66E-294	0.00557209	2.16E-175	0.994095938	0.000331968
HLA-DQA2	5345	9.30E-103	0.01197295	1.21E-85	0.9835284	0.004498653
HSPA1L	5837	2.44E-57	0.00249477	3.17E-73	0.254142326	0.7433629
IL23R	4234	0	7.37E-79	0	1	3.22E-45
LILRB3	5967	6.23E-134	0.06756285	8.10E-141	0.918001065	0.01443609
MAPK14	4345	0	2.00E-07	0	0.022823	0.9771768
MICA	3487	6.32E-36	2.75E-05	8.22E-64	0.144419507	0.855553
MICB	6498	4.68E-63	0.13818637	6.08E-74	0.849662271	0.01215136
MPIG6B	4543	0	0.00280414	0	0.996948415	0.000247442
TNXB	5349	0	4.98E-09	0	0.735989895	0.2640101
VARS1	4591	9.52E-48	0.86206095	1.24E-52	0.038984424	0.09895463

Number of single nucleotide polymorphisms (nsnp),posterior probability of pypotheses (PPH),each identifier for the exposure factor being investigated, which in our study refers to specific plasma proteins (Protein).

### Phenomewide MR analysis

We sourced data on gene-trait associations from the GWAS Catalog accessible at: https://gwas.mrcieu.ac.uk/. Our analysis focused on examining the link between gene variants and traits. We utilized cis-pQTLs to understand these relationships and conducted thorough searches to pinpoint studies that reported statistically significant genetic associations (*P*< 0.001) with specific traits. Our study incorporated a detailed PheWAS analysis to investigate how the top six proteins that colocalize—HSPA1L, MICA, COL11A2, MAPK14, AIF1, and ATF6B were connected to We discovered that these proteins not only contribute to targeting AS but also significantly impact the development of other diseases. For example, AIF1, MICA, ATF6B, and HSPA1L were found to be associated with an increased risk of rheumatoid arthritis (RA), while AIF1 was also associated with an increased risk of atopic dermatitis. On the other hand, MAPK14, ATF6B, and COL11A2 were associated with a lower risk of both rheumatoid arthritis and primary sclerosing cholangitis (refer to **Supplementary Table S2**). exploration enables us to deepen our understanding of how specific genetic variations can influence disease mechanisms, providing critical insights for genetic research and biomedical applications beyond just treating AS.

### Drug validation

Our research identified MAPK14 as a potential genetic drug target and biomarker in AS. By analyzing the DGIdb database, we discovered a link between MAPK14 and the drug pirfenidone, which is currently under clinical investigation. Interestingly, MAPK14 is also associated with three FDA-approved drugs: dasatinib, doxorubicin, and sorafenib. Although our study did not directly involve pharmaceutical action on AS, these findings provided new prospects for the treatment of AS and related diseases (**Supplementary Table S3**).

### Molecular docking

**Figure 4:**
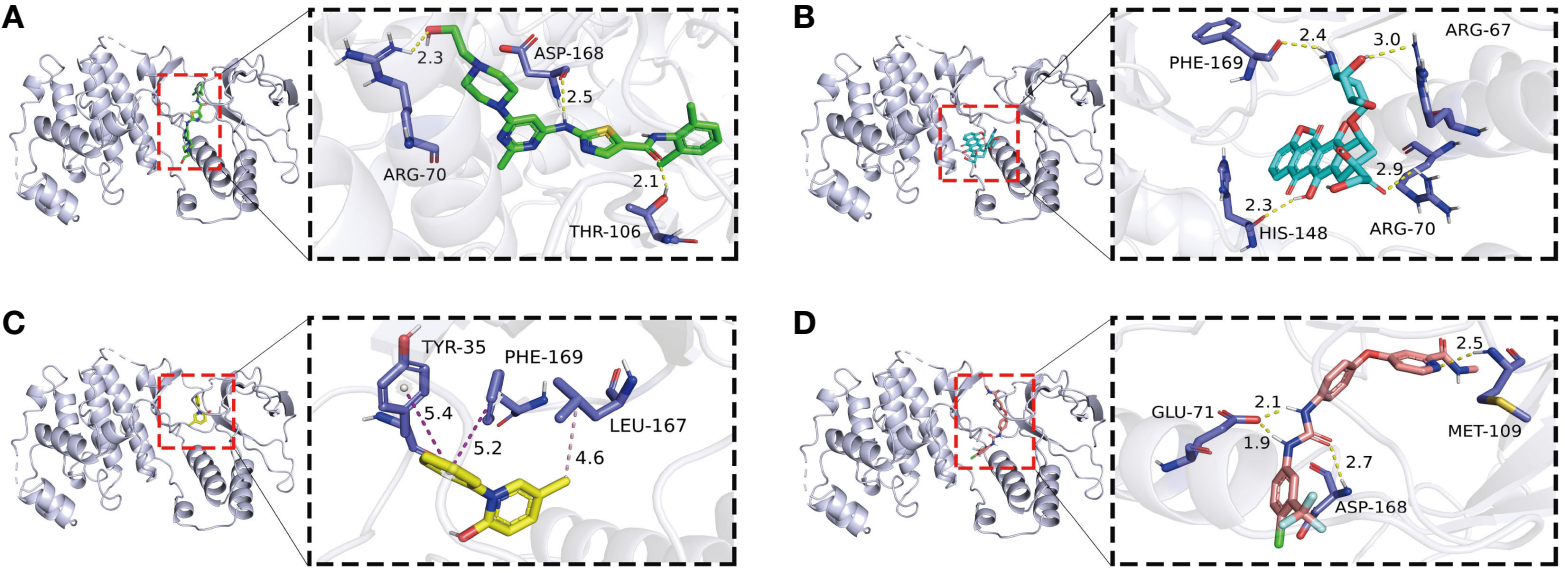
Molecular docking analysis of four compounds with a target protein (MAPK14): **(A-D)** display the binding interactions between MAPK14 and four different ligands: dasatinib **(A)**, doxorubicin **(B)**, pirfenidone **(C)**, and sorafenib **(D)**. Each panel illustrates the protein in a ribbon diagram with the ligand in a stick model, zooming in on the binding site. The dashed lines represent potential hydrogen bonds or hydrophobic contacts with key amino acids, with distances measured in angstroms.

**Figure 5:**
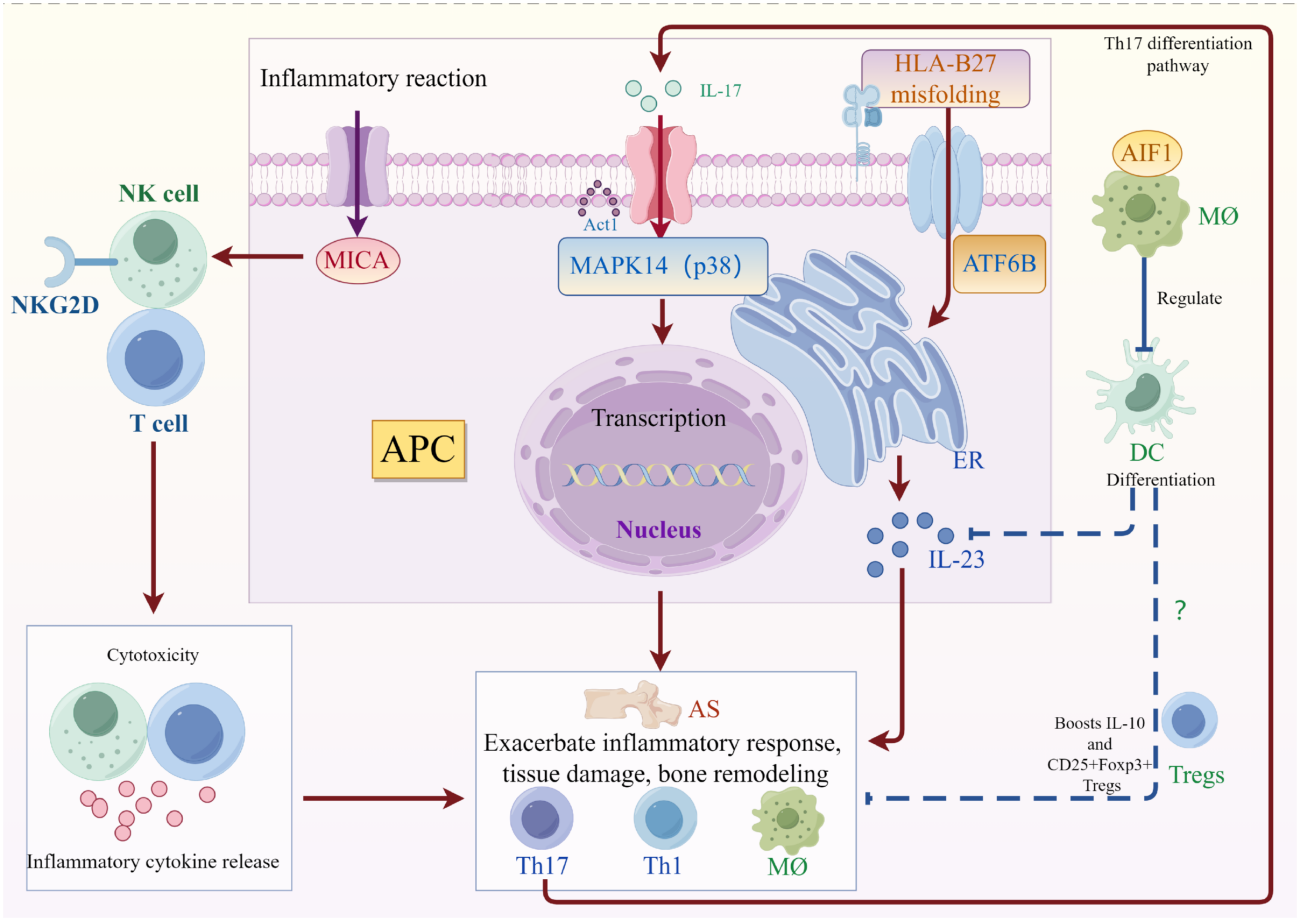
Summary figure of molecular and cellular mechanisms in ankylosing spondylitis pathogenesis: This figure illustrates the roles of four key targets in the pathogenesis of AS: MICA, MAPK14, ATF6B, and AIF1. MICA binds to the NKG2D receptor on NK cells and T cells, initiating cytotoxicity and inflammatory cytokine release. MAPK14, also known as p38, is activated by Act1, triggering transcriptional changes that promote inflammation. ATF6B is involved in HLA-B27 misfolding, contributing to inflammation, but also suppresses ER stress from HLA-B27 variants. AIF1, expressed in macrophages, regulates immune responses and is associated with the differentiation of DCs, further might drive Tregs to regulate immune response, and boost IL-10.

In the realm of molecular docking (MD) investigations, the concept of binding energy serves as a fundamental metric for assessing ligand-receptor interactions. A binding energy less than 0 kcal/mol is indicative of a spontaneous binding process, confirming the thermodynamic feasibility of the ligand associating with the receptor. Specifically, a binding energy threshold of -7.2 kcal/mol is often utilized as a benchmark for strong molecular interactions, characterized by elevated affinity and specificity towards the receptor. In our analysis, we evaluated the docking profiles of pirfenidone, dasatinib, doxorubicin, and sorafenib with the kinase domain of MAPK14. The results demonstrated binding energies of -9.7 kcal/mol, -9.0 kcal/mol, -8.5 kcal/mol, and -12 kcal/mol, respectively. All values significantly surpass the -7.2 kcal/mol threshold, suggesting robust binding affinities (see **Figure 4**, **Supplementary Table S4** for details). Concurrently, we employed the methodology of molecular docking to validate the co-crystallized ligand with MAPK14 (**Supplementary Figure S1**). The RMSD (Root Mean Square Deviation) value between the co-crystal ligand and MAPK14 before and after docking is 0.321Å, which is less than 2Å, indicating that the molecular docking methodology has been validated. These findings imply a high potential for these compounds to inhibit MAPK14 activity effectively, which may be therapeutically beneficial in modulating pathways regulated by this kinase in AS (**Figure 5**).

## Discussion

To date, the treatment of ankylosing spondylitis (AS) continues to pose a considerable clinical challenge within the realm of medicine (41). The discovery and development of innovative pharmaceuticals for AS are hindered by high costs and a limited number of approved therapeutic agents, which are often associated with severe adverse reactions or patient intolerance (42). Recent advances in human genetics have led to the identification of pharmacological targets that are pertinent for AS treatment (43). Some Food and Drug Administration (FDA) approved medications for AS-target immune system inflammation, such as tumor necrosis factor inhibitors and interleukin-17 inhibitors, to alleviate symptoms (44). In this study, plasma proteomic data served as valuable resources for identifying potential drug targets. Using mendelian randomization (MR) analysis with matched AS-GWAS (Genome-wide association study) samples, we established causal relationships between genetic plasma proteomic variations associated with AS risk and the expression of specific proteins following the methods described in previous literature (45). Simultaneously, the Phenome-wide Association Study (PheWAS) explores the connections between these proteins and a broad spectrum of phenotypic traits, enhancing our understanding of their roles across various medical conditions (46). This approach increases the likelihood of successful drug development while reducing development costs. Ultimately, we identified 18 promising proteins for drug research, including AGER, AIF1, ATF6B, C4A, CFB, CLIC1, COL11A2, ERAP1, HLA-DQA2, HSPA1L, IL23R, LILRB3, MAPK14, MICA, MICB, MPIG6B, TNXB and VARS1. Among them, AIF1 and IL23R are associated with decreased risk of ankylosing spondylitis; MICA, MAPK14, and ATF6B are linked to increased risk of ankylosing spondylitis.

C4 and C4A, pivotal proteins within the human immune system, are encoded by distinct genes and categorized as C4 isotypes (47). They activate the complement pathway, crucial for pathogen clearance, and C4A is implicated in autoimmune disease risk due to its regulatory role in autoimmune responses (48). An *in vitro* studies suggest that AS pathogenesis is closely linked to complement activation, aligning with our findings (49).

Endoplasmic reticulum aminopeptidase 1 (ERAP1) plays a vital role in clarifying important biological pathways in AS pathogenesis, mainly linked to human leukocyte antigen B27 (HLA-B27) positive cases, by influencing peptides binding to class I molecules of the major histocompatibility complex (MHC) (50). ERAP1’s interaction with HLA-B27 influences the peptide processing crucial for disease susceptibility in AS. ERAP1 polymorphism correlates with AS severity and progression (51–53). Research by Chen et al. demonstrated that reducing ERAP1 levels decreases the surface presence of free HLA-B27 heavy chains (FHC) on antigen-presenting cells. This reduction lowers the activation of KIR3DL2, a critical immune receptor, consequently diminishing interleukin-2 (IL-2) production (54). These changes resulted in an expansion of Th17 cells and decreased IL-17A secretion in CD4^+^ T cells among patients with AS. The study suggested that the activity of ERAP1 determines the surface expression of HLA-B27 FHCs and may potentially facilitate the Th17 response through the interaction between HLA-B27 FHCs and KIR3DL2. Therefore, the activity of ERAP1 appears to be crucial in regulating the Th17 cell response and IL-2 production in AS. Our research work aligned with these results, suggesting that proteins C4A and ERAP1 may exert an influence on AS development by modulating the immune response, especially within the inflammatory pathways.

MAPK14 (mitogen-activated protein kinase 14), also known as p38 MAPK, plays a significant role in inflammation and immune response, linked to AS through gene polymorphisms near the MAPK14 locus (55, 56). Elevated gene expression levels of Myd88 (myeloid differentiation primary response 88), NF-kB (nuclear factor-kappa B) and MAPK14 in AS patients, compared to controls, were reported by Roozbehkia et al. (57). Although this work did not identify any FDA-approved therapeutics for AS that target MAPK14, molecular docking (MD) analysis was implemented to substantiate these findings. Our analysis utilized the Drug-Gene Interaction database (DGIdb) to ascertain pharmaceuticals associated with MAPK14, identifying pirfenidone, dasatinib, doxorubicin, and sorafenib. These agents demonstrated potent binding affinity to MAPK14, suggesting their potential efficacy in mitigating the enzymatic activity of MAPK14, thereby offering prospective therapeutic benefits in the management of AS. Pirfenidone, approved by the FDA for the treatment of idiopathic pulmonary fibrosis (IPF), shows promising action on AS. Pirfenidone possesses anti-inflammatory and antifibrotic properties; its mechanisms include inhibition of TGF-β-induced fibroblast proliferation and suppression of inflammatory mediator production (58). Given the chronic inflammation and potential fibrosis in the pathological process of AS, including new bone formation (59), pirfenidone may have potential therapeutic utility in the treatment of AS. Due to the critical role of MAPK14 in regulating inflammation, inhibitors targeting this protein may lead to adverse effects, such as immunosuppression (60). Similarly, the immunomodulatory functions of activating transcription factor 6B (ATF6B), and AIF1 suggest that targeting these proteins could disrupt crucial immune processes, such as autophagy and apoptosis (61, 62). Future research will continue to assess these side effects and rigorously test them in preclinical studies to ensure patient safety.

In a summary, MAPK can be activated by various inflammatory cytokines, chemokines, and pattern recognition receptors, including IL-17, may affect cell proliferation, differentiation, and apoptosis in AS through cascades of signal transduction (63). The major histocompatibility complex class I (MHC) chain-related protein A (MICA) protein, which is activated in response to stress in natural killer (NK) cells, γδ T cells, and other immune cells, binds to the NK receptor group 2 member D (NKG2D) receptor, triggering cytotoxicity and the release of inflammatory cytokines by NK cells and T cells. An imbalance in this pathway may increase the risk of AS (64). Unconventional HLA-B27 variants may trigger endoplasmic reticulum stress (ERS) through homodimerization, and ATF6B, a transmembrane protein with low transcriptional activity in the ER, may suppress ER stress activation and inflammation (65, 66). Allograft inflammatory factor 1 (AIF1), a calcium-binding protein, is involved in phagocytosis, membrane ruffling, and F-actin remodeling, and is associated with macrophage activation and various diseases (67). Research shows that knocking down Allograft Inflammatory Factor 1 (AIF1) suppresses antigen-specific CD4+ T cell proliferation, increases IL-10 production, and expands CD25+Foxp3+ regulatory T cell subsets, which inhibits inflammation (68). This observation is consistent with the results of our work. AIF1 might play a significant role in AS by regulating the differentiation and function of dendritic cells (**Figure 5**). Biological networks are complex, and it is postulated that in AS, MAPK14 and ATF6B may be interconnected through cellular and endoplasmic reticulum stress responses, while MICA and AIF1 may be associated with immune responses and inflammation.

Interleukin-12 (IL-12) may play a role in the pathogenesis of AS. Excessive IL-12 activity can trigger abnormal immune responses, potentially contributing to the development and worsening of AS (69). IL-12/23 monoclonal antibody (Ustekinumab) is currently approved to treat AS to alleviate symptoms (70). More research is needed to clarify the exact role of IL-12 in the pathogenic mechanisms of AS.

Recent studies highlight Th17 cells’ adaptive gene expression, influencing their pathogenic potential in AS (71, 72). These cells, which can co-express RORγt and TBX21, are implicated in autoimmune pathologies, while others produce cytokines targeting specific pathogens (73, 74). Elevated Th17 activity correlates with increased inflammatory cytokine production, contributing to bone damage in AS (75). IL23R polymorphisms also relate to disease susceptibility, influencing IL-17 production (76–78). Our findings support the development of multitarget drugs to inhibit Th17 differentiation and reduce pro-inflammatory cytokine production as a therapeutic strategy for AS.

This study identified genetic associations between circulating proteins and AS using large-scale MR analysis, revealing novel pathways in AS pathogenesis. The results were robust, adhering to stringent instrumental variable criteria. Additionally, a PheWAS analysis assessed the safety of these proteins as drug targets, aiming to mitigate severe side effects.

However, limitations exist. The proteins studied were derived from whole blood and may not reflect tissue-specific processes in AS. Moreover, the magnetic resonance approach presumes uniform drug efficacy across individuals, which contrasts with variable clinical responses. The findings predominantly involve European populations, highlighting the need for more diverse studies to enhance generalizability and understand protein-specific mechanisms in AS. Due to limitations, *in vitro* and *in vivo* experiments may be constrained. We plan to include them in future research or engage in multicenter laboratory collaboration.

In conclusion, this research highlights the utility of MR in identifying potential drug targets or biomarkers for AS, implicating proteins involved in Th17 differentiation in AS pathogenesis. These proteins could serve as early screening tools or therapeutic targets. Future research is necessary to confirm these potential applications.

## Data availability statement

The original contributions presented in the study are included in the article/**Supplementary Material**. Further inquiries can be directed to the corresponding author.

## Ethics statement

All the studies involved to complement this study were approved by relevant ethics committees. All participants had provided the written informed consent in these original studies respectively. for the studies involving humans because All the studies involved to complement this study were approved by relevant ethics committees. All participants had provided the written informed consent in these original studies respectively. The studies were conducted in accordance with the local legislation and institutional requirements. The participants provided their written informed consent to participate in this study.

## Author contributions

YZ: Conceptualization, Funding acquisition, Investigation, Methodology, Project administration, Resources, Supervision, Writing – original draft, Writing – review & editing. WL: Conceptualization, Investigation, Methodology, Project administration, Resources, Supervision, Writing – review & editing. JL: Conceptualization, Data curation, Formal Analysis, Investigation, Methodology, Resources, Writing – original draft. HZ: Writing – review & editing, Writing – original draft, Resources, Methodology, Investigation, Formal Analysis, Data curation.

## Supplementary material

Supplementary Figure 1 Molecular docking Analysis of MAPK14 protein with a co-crystal ligand. The left panel shows the overall structure of the MAPK14 protein. The right panel illustrates the specific interactions between the co-crystal ligand and key amino acid residues in the MAPK14 protein. The panel represents the protein in a ribbon diagram, with the ligand depicted in a stick model, focusing on the binding site. The yellow dashed lines represent potential hydrogen bond contacts with key amino acids, with distances given in angstroms. quantitative trait loci (pQTLs), Instrumental variables (IVs), single nucleotide polymorphism (SNP), linkage disequilibrium (LD), mendelian randomization (MR), Wald ratio (WR), ankylosing spondylitis (AS), gene ontology (GO), Kyoto encyclopedia of genes and genomes (KEGG), Drug gene interaction database (DGIdb), Phenome-wide association study (PheWAS),Molecular docking (MD), major histocompatibility complex class I (MHC) chain-related protein A (MICA), mitogen-activated protein kinase 14 (MAPK14), activating transcription factor 6B (ATF6B), Allograft inflammatory factor 1 (AIF1), NK receptor group 2 member D (NKG2D), natural killer (NK), Apoptosis-associated speck-like protein containing a CARD (Act1), endoplasmic reticulum (ER), human leukocyte antigen B27 (HLA-B27), dendritic cells (DCs), regulatory T cells (Tregs), Macrophages (MØ), Interleukin 17 (IL-17), Interleukin 23 (IL-23), Interleukin 10 (IL-10), T-helper (Th) cells, Forkhead box protein P (FoxP), Antigen presenting cell (APC).

## References

[1] Yang H Chen Y Xu W Shao M Deng J Xu S. Epigenetics of ankylosing spondylitis: Recent developments. Int J Rheum Dis. (2021) 24:487–93. doi: 10.1111/1756-185X.14080 33608999

[2] Kwon SR Kim TH Kim TJ Park W Shim SC. The epidemiology and treatment of ankylosing spondylitis in Korea. J Rheum Dis. (2022) 29:193–9. doi: 10.4078/jrd.22.0023 PMC10351411 37476425

[3] Jung SM Kim WU. Targeted immunotherapy for autoimmune disease. Immune Netw. (2022) 22:e9. doi: 10.4110/in.2022.22.e9 35291650 PMC8901705

[4] Ward MM Deodhar A Gensler LS Dubreuil M Yu D Khan MA. 2019 Update of the American college of rheumatology/spondylitis association of America/spondyloarthritis research and treatment network recommendations for the treatment of ankylosing spondylitis and nonradiographic axial spondyloarthritis. Arthritis Care Res (Hoboken). (2019) 71:1285–99. doi: 10.1002/acr.24025 PMC6764857 31436026

[5] Berdigaliyev N Aljofan M. An overview of drug discovery and development. Future Med Chem. (2020) 12:939–47. doi: 10.4155/fmc-2019-0307 32270704

[6] Jin M Liu P Qi G. Exploring potential biomarkers of early thymoma based on serum proteomics. Protein Pept Lett. (2023) 31:74–83. doi: 10.2174/0109298665275655231103105924 38053354

[7] Wu J Tang B Tang Y. Allele-specific genome targeting in the development of precision medicine. Theranostics. (2020) 10:3118–37. doi: 10.7150/thno.43298 PMC7053192 32194858

[8] Carss KJ Deaton AM Del Rio-Espinola A Diogo D Fielden M Kulkarni DA. Using human genetics to improve safety assessment of therapeutics. Nat Rev Drug Discovery. (2023) 22:145–62. doi: 10.1038/s41573-022-00561-w 36261593

[9] Chauquet S Zhu Z O’Donovan MC Walters JTR Wray NR Shah S. Association of antihypertensive drug target genes with psychiatric disorders: A Mendelian randomization study. JAMA Psychiatry. (2021) 78:623–31. doi: 10.1001/jamapsychiatry.2021.0005 PMC7948097 33688928

[10] Bakker MK van Straten T Chong M Paré G Gill D Ruigrok YM. Anti-epileptic drug target perturbation and intracranial aneurysm risk: Mendelian randomization and colocalization study. Stroke. (2023) 54:208–16. doi: 10.1161/STROKEAHA.122.040598 PMC9794136 36300369

[11] Yang C Farias FHG Ibanez L Suhy A Sadler B Fernandez MV. Genomic atlas of the proteome from brain, CSF and plasma prioritizes proteins implicated in neurological disorders. Nat Neurosci. (2021) 24:1302–12. doi: 10.1038/s41593-021-00886-6 PMC8521603 34239129

[12] Sun BB Maranville JC Peters JE Stacey D Staley JR Blackshaw J. Genomic atlas of the human plasma proteome. Nature. (2018) 558:73–9. doi: 10.1038/s41586-018-0175-2 PMC6697541 29875488

[13] Suhre K Arnold M Bhagwat AM Cotton RJ Engelke R Raffler J. Connecting genetic risk to disease end points through the human blood plasma proteome. Nat Commun. (2017) 8:14357. doi: 10.1038/ncomms14357 28240269 PMC5333359

[14] Folkersen L Fauman E Sabater-Lleal M Strawbridge RJ Frånberg M Sennblad B. Mapping of 79 loci for 83 plasma protein biomarkers in cardiovascular disease. PloS Genet. (2017) 13:e1006706. doi: 10.1371/journal.pgen.1006706 28369058 PMC5393901

[15] Yao C Chen G Song C Keefe J Mendelson M Huan T. Genome-wide mapping of plasma protein QTLs identifies putatively causal genes and pathways for cardiovascular disease. Nat Commun. (2018) 9:3268. doi: 10.1038/s41467-018-05512-x 30111768 PMC6093935

[16] Emilsson V Ilkov M Lamb JR Finkel N Gudmundsson EF Pitts R. Co-regulatory networks of human serum proteins link genetics to disease. Science. (2018) 361:769–73. doi: 10.1126/science.aaq1327 PMC6190714 30072576

[17] Zheng J Haberland V Baird D Walker V Haycock PC Hurle MR. Phenome-wide Mendelian randomization mapping the influence of the plasma proteome on complex diseases. Nat Genet. (2020) 52:1122–31. doi: 10.1038/s41588-020-0682-6 PMC7610464 32895551

[18] Yang M Su Y Xu K Wan X Xie J Liu L. Iron, copper, zinc and magnesium on rheumatoid arthritis: a two-sample Mendelian randomization study. Int J Environ Health Res. (2023) 30:1–14. doi: 10.1080/09603123.2023.2274377 37903459

[19] Bottigliengo D Foco L Seibler P Klein C König IR Del Greco MF. A Mendelian randomization study investigating the causal role of inflammation on Parkinson’s disease. Brain. (2022) 145:3444–53. doi: 10.1093/brain/awac193 PMC9586538 35656776

[20] Gudicha DW Schmittmann VD Vermunt JK. Statistical power of likelihood ratio and Wald tests in latent class models with covariates. Behav Res Methods. (2017) 49:1824–37. doi: 10.3758/s13428-016-0825-y PMC5628195 28039681

[21] Fridley BL Iversen E Tsai YY Jenkins GD Goode EL Sellers TA. A latent model for prioritization of SNPs for functional studies. PloS One. (2011) 6:e20764. doi: 10.1371/journal.pone.0020764 21687685 PMC3110798

[22] Armstrong RA. When to use the Bonferroni correction. Ophthalmic Physiol Opt. (2014) 34:502–8. doi: 10.1111/opo.12131 24697967

[23] Tang D Chen M Huang X Zhang G Zeng L Zhang G. SRplot: A free online platform for data visualization and graphing. PloS One. (2023) 18:e0294236. doi: 10.1371/journal.pone.0294236 37943830 PMC10635526

[24] Burgess S Small DS Thompson SG. A review of instrumental variable estimators for Mendelian randomization. Stat Methods Med Res. (2017) 26:2333–55. doi: 10.1177/0962280215597579 PMC5642006 26282889

[25] Hukku A Pividori M Luca F Pique-Regi R Im HK Wen X. Probabilistic colocalization of genetic variants from complex and molecular traits: promise and limitations. Am J Hum Genet. (2021) 108:25–35. doi: 10.1016/j.ajhg.2020.11.012 33308443 PMC7820626

[26] Su J Zhou W Yuan H Wang H Zhang H. Identification and functional analysis of novel biomarkers in adenoid cystic carcinoma. Cell Mol Biol (Noisy-le-grand). (2023) 69:203–7. doi: 10.14715/cmb/2023.69.6.31 37605568

[27] Lin J Zhou J Xu Y. Potential drug targets for multiple sclerosis identified through Mendelian randomization analysis. Brain. (2023) 146:3364–72. doi: 10.1093/brain/awad070 PMC10393411 36864689

[28] Chen Y Li C Cheng S Pan C Zhang H Zhang J. The causal relationships between sleep-related phenotypes and body composition: A Mendelian randomized study. J Clin Endocrinol Metab. (2022) 107:e3463–73. doi: 10.1210/clinem/dgac234 35435981

[29] Yang R Chen H Xing L Wang B Hu M Ou X. Hypoxia-induced circWSB1 promotes breast cancer progression through destabilizing p53 by interacting with USP10. Mol Cancer. (2022) 21:88. doi: 10.1186/s12943-022-01567-z 35351136 PMC8961958

[30] Yuan S Sun J Lu Y Xu F Li D Jiang F. Health effects of milk consumption: phenome-wide Mendelian randomization study. BMC Med. (2022) 20:455. doi: 10.1186/s12916-022-02658-w 36424608 PMC9694907

[31] Liu X Tian D Li C Tang B Wang Z Zhang R. GWAS Atlas: an updated knowledgebase integrating more curated associations in plants and animals. Nucleic Acids Res. (2023) 51:D969–76. doi: 10.1093/nar/gkac924 PMC9825481 36263826

[32] Kamat MA Blackshaw JA Young R Surendran P Burgess S Danesh J. PhenoScanner V2: an expanded tool for searching human genotype-phenotype associations. Bioinformatics. (2019) 35:4851–3. doi: 10.1093/bioinformatics/btz469 PMC6853652 31233103

[33] Freshour SL Kiwala S Cotto KC Coffman AC McMichael JF Song JJ. Integration of the Drug-Gene Interaction Database (DGIdb 4.0) with open crowdsource efforts. Nucleic Acids Res. (2021) 49:D1144–51. doi: 10.1093/nar/gkaa1084 PMC7778926 33237278

[34] Wang J Kang Z Liu Y Li Z Liu Y Liu J. Identification of immune cell infiltration and diagnostic biomarkers in unstable atherosclerotic plaques by integrated bioinformatics analysis and machine learning. Front Immunol. (2022) 13:956078. doi: 10.3389/fimmu.2022.956078 36211422 PMC9537477

[35] Prestegard JH. A perspective on the PDB’s impact on the field of glycobiology. J Biol Chem. (2021) 296:100556. doi: 10.1016/j.jbc.2021.100556 33744289 PMC8058564

[36] Seeliger D de Groot BL. Ligand docking and binding site analysis with PyMOL and Autodock/Vina. J Comput Aided Mol Des. (2010) 24:417–22. doi: 10.1007/s10822-010-9352-6 PMC2881210 20401516

[37] Wang Y Xiao J Suzek TO Zhang J Wang J Zhou Z. PubChem’s bioAssay database. Nucleic Acids Res. (2012) 40:D400–12. doi: 10.1093/nar/gkr1132 PMC3245056 22140110

[38] O’Boyle NM Morley C Hutchison GR. Pybel: a Python wrapper for the OpenBabel cheminformatics toolkit. Chem Cent J. (2008) 2:5. doi: 10.1186/1752-153X-2-5 18328109 PMC2270842

[39] Harini M Kavitha K Prabakaran V Krithika A Dinesh S Rajalakshmi A. Identification of apigenin-4’-glucoside as bacterial DNA gyrase inhibitor by QSAR modeling, molecular docking, DFT, molecular dynamics, and in *vitro* confirmation studies. J Mol Model. (2024) 30:22. doi: 10.1007/s00894-023-05813-z 38170229

[40] Govender N Zulkifli NS Badrul Hisham NF Ab Ghani NS Mohamed-Hussein ZA. Pea eggplant (Solanum torvum Swartz) is a source of plant food polyphenols with SARS-CoV inhibiting potential. PeerJ. (2022) 10:e14168. doi: 10.7717/peerj.14168 36518265 PMC9744172

[41] Fattorini F Gentileschi S Cigolini C Terenzi R Pata AP Esti L. Axial spondyloarthritis: one year in review 2023. Clin Exp Rheumatol. (2023) 41:2142–50. doi: 10.55563/clinexprheumatol/9fhz98 37965699

[42] Drosos AA Venetsanopoulou AI Voulgari PV. Axial Spondyloarthritis: Evolving concepts regarding the disease’s diagnosis and treatment. Eur J Intern Med. (2023) 117:21–7. doi: 10.1016/j.ejim.2023.06.026 37414646

[43] Kenyon M Maguire S Rueda Pujol A O’Shea F McManus R. The genetic backbone of ankylosing spondylitis: how knowledge of genetic susceptibility informs our understanding and management of disease. Rheumatol Int. (2022) 42:2085–95. doi: 10.1007/s00296-022-05174-5 PMC9548471 35939079

[44] Chen Y Liu S Gong W Guo P Xue F Zhou X. Protein-centric omics integration analysis identifies candidate plasma proteins for multiple autoimmune diseases. Hum Genet. (2023). doi: 10.1007/s00439-023-02627-0 PMC11485194 38143258

[45] Gui J Meng L Huang D Wang L Yang X Ding R. Identification of novel proteins for sleep apnea by integrating genome-wide association data and human brain proteomes. Sleep Med. (2023) 114:92–9. doi: 10.1016/j.sleep.2023.12.026 38160582

[46] Schifferli JA Paccaud JP. Two isotypes of human C4, C4A and C4B have different structure and function. Complement Inflamm. (1989) 6:19–26. doi: 10.1159/000463068 2650988

[47] Coss SL Zhou D Chua GT Aziz RA Hoffman RP Wu YL. The complement system and human autoimmune diseases. J Autoimmun. (2023) 137:102979. doi: 10.1016/j.jaut.2022.102979 36535812 PMC10276174

[48] Yang C Ding P Wang Q Zhang L Zhang X Zhao J. Inhibition of complement retards ankylosing spondylitis progression. Sci Rep. (2016) 6:34643. doi: 10.1038/srep34643 27698377 PMC5048143

[49] Fatica M D’Antonio A Novelli L Triggianese P Conigliaro P Greco E. How has molecular biology enhanced our undertaking of axSpA and its management. Curr Rheumatol Rep. (2023) 25:12–33. doi: 10.1007/s11926-022-01092-4 36308677 PMC9825525

[50] Saad MA Abdul-Sattar AB Abdelal IT Baraka A. Shedding light on the role of ERAP1 in axial spondyloarthritis. Cureus. (2023) 15:e48806. doi: 10.7759/cureus.48806 38024089 PMC10645460

[51] Evans DM Spencer CC Pointon JJ Su Z Harvey D Kochan G. Interaction between ERAP1 and HLA-B27 in ankylosing spondylitis implicates peptide handling in the mechanism for HLA-B27 in disease susceptibility. Nat Genet. (2011) 43:761–7. doi: 10.1038/ng.873 PMC3640413 21743469

[52] Küçükşahin O Ateş A Türkçapar N Törüner M Turgay M Duman T. Association between single nucleotide polymorphisms in prospective genes and susceptibility to ankylosing spondylitis and inflammatory bowel disease in a single centre in Turkey. Turk J Gastroenterol. (2016) 27:317–24. doi: 10.5152/tjg.2016.15466 27458846

[53] Chen R Yao L Meng T Xu W. The association between seven ERAP1 polymorphisms and ankylosing spondylitis susceptibility: a meta-analysis involving 8,530 cases and 12,449 controls. Rheumatol Int. (2012) 32:909–14. doi: 10.1007/s00296-010-1712-y 21229357

[54] Chen L Ridley A Hammitzsch A Al-Mossawi MH Bunting H Georgiadis D. Silencing or inhibition of endoplasmic reticulum aminopeptidase 1 (ERAP1) suppresses free heavy chain expression and Th17 responses in ankylosing spondylitis. Ann Rheum Dis. (2016) 75:916–23. doi: 10.1136/annrheumdis-2014-206996 PMC4853590 26130142

[55] Bonney EA. Mapping out p38MAPK. Am J Reprod Immunol. (2017) 77:10. doi: 10.1111/aji.12652 PMC5527295 28194826

[56] Costantino F Talpin A Said-Nahal R Leboime A Zinovieva E Zelenika D. A family-based genome-wide association study reveals an association of spondyloarthritis with MAPK14. Ann Rheum Dis. (2017) 76:310–4. doi: 10.1136/annrheumdis-2016-209449 27461236

[57] Roozbehkia M Mahmoudi M Aletaha S Rezaei N Fattahi MJ Jafarnezhad-Ansariha F. The potent suppressive effect of β-d-mannuronic acid (M2000) on molecular expression of the TLR/NF-kB Signaling Pathway in ankylosing spondylitis patients. Int Immunopharmacol. (2017) 52:191–6. doi: 10.1016/j.intimp.2017.08.018 28938189

[58] Man RK Gogikar A Nanda A Janga LSN Sambe HG Yasir M. A comparison of the effectiveness of nintedanib and pirfenidone in treating idiopathic pulmonary fibrosis: A systematic review. Cureus. (2024) 16:e54268. doi: 10.7759/cureus.54268 38500898 PMC10945152

[59] Liu Z Cai M Ke H Deng H Ye W Wang T. Fibroblast insights into the pathogenesis of ankylosing spondylitis. J Inflammation Res. (2023) 16:6301–17. doi: 10.2147/JIR.S439604 PMC10750494 38149115

[60] Lo U Selvaraj V Plane JM Chechneva OV Otsu K Deng W. p38α (MAPK14) critically regulates the immunological response and the production of specific cytokines and chemokines in astrocytes. Sci Rep. (2014) 4:7405. doi: 10.1038/srep07405 25502009 PMC4264013

[61] Zhao YY Yan DJ Chen ZW. Role of AIF-1 in the regulation of inflammatory activation and diverse disease processes. Cell Immunol. (2013) 284:75–83. doi: 10.1016/j.cellimm.2013.07.008 23948156

[62] Rozpedek W Nowak A Pytel D Diehl JA Majsterek I. Molecular basis of human diseases and targeted therapy based on small-molecule inhibitors of ER stress-induced signaling pathways. Curr Mol Med. (2017) 17:118–32. doi: 10.2174/1566524017666170306122643 28266275

[63] Arthur JS Ley SC. Mitogen-activated protein kinases in innate immunity. Nat Rev Immunol. (2013) 13:679–92. doi: 10.1038/nri3495 23954936

[64] Fernández-Torres J Zamudio-Cuevas Y Ruiz-Dávila X López-Macay A Martínez-Flores K. MICA and NLRP3 gene polymorphisms interact synergistically affecting the risk of ankylosing spondylitis. Immunol Res. (2024) 72:119–27. doi: 10.1007/s12026-023-09419-8 37665559

[65] Guiliano DB Fussell H Lenart I Tsao E Nesbeth D Fletcher AJ. Endoplasmic reticulum degradation-enhancing α-mannosidase-like protein 1 targets misfolded HLA-B27 dimers for endoplasmic reticulum-associated degradation. Arthritis Rheumatol. (2014) 66:2976–88. doi: 10.1002/art.38809 PMC4399817 25132672

[66] Forouhan M Mori K Boot-Handford RP. Paradoxical roles of ATF6α and ATF6β in modulating disease severity caused by mutations in collagen X. Matrix Biol. (2018) 70:50–71. doi: 10.1016/j.matbio.2018.03.004 29522813 PMC6090092

[67] De Leon-Oliva D Garcia-Montero C Fraile-Martinez O Boaru DL García-Puente L Rios-Parra A. AIF1: function and connection with inflammatory diseases. Biol (Basel). (2023) 12:694. doi: 10.3390/biology12050694 PMC10215110 37237507

[68] Elizondo DM Andargie TE Yang D Kacsinta AD Lipscomb MW. Inhibition of allograft inflammatory factor-1 in dendritic cells restrains CD4+ T cell effector responses and induces CD25+Foxp3+ T regulatory subsets. Front Immunol. (2017) 8:1502. doi: 10.3389/fimmu.2017.01502 29167673 PMC5682305

[69] Fiehn C. Biologikatherapie von rheumatoider Arthritis und Spondyloarthritiden [Treatment of rheumatoid arthritis and spondylarthritis with biologics]. Internist (Berl). (2022) 63:135–42. doi: 10.1007/s00108-021-01248-x PMC8759427 35029702

[70] Tahir H. Therapies in ankylosing spondylitis-from clinical trials to clinical practice. Rheumatol (Oxford). (2018) 57:vi23–8. doi: 10.1093/rheumatology/key152 PMC6238222 30445480

[71] Shi Y Wei B Li L Wang B Sun M. Th17 cells and inflammation in neurological disorders: Possible mechanisms of action. Front Immunol. (2022) 13:932152. doi: 10.3389/fimmu.2022.932152 35935951 PMC9353135

[72] Simone D Stingo A Ciccia F. Genetic and environmental determinants of T helper 17 pathogenicity in spondyloarthropathies. Front Genet. (2021) 12:703242. doi: 10.3389/fgene.2021.703242 34630512 PMC8492997

[73] Zeng J Li M Zhao Q Chen M Zhao L Wei S. Small molecule inhibitors of RORγt for Th17 regulation in inflammatory and autoimmune diseases. J Pharm Anal. (2023) 13:545–62. doi: 10.1016/j.jpha.2023.05.009 PMC10334362 37440911

[74] Zielinski CE Mele F Aschenbrenner D Jarrossay D Ronchi F Gattorno M. Pathogen-induced human TH17 cells produce IFN-γ or IL-10 and are regulated by IL-1β. Nature. (2012) 484:514–8. doi: 10.1038/nature10957 22466287

[75] Zhu W He X Cheng K Zhang L Chen D Wang X. Ankylosing spondylitis: etiology, pathogenesis, and treatments. Bone Res. (2019) 7:22. doi: 10.1038/s41413-019-0057-8 31666997 PMC6804882

[76] Wellcome Trust Case Control Consortium. Genome-wide association study of 14,000 cases of seven common diseases and 3,000 shared controls. Nature. (2007) 447:661–78. doi: 10.1038/nature05911 PMC2719288 17554300

[77] Vecellio M Cohen CJ Roberts AR Wordsworth PB Kenna TJ. RUNX3 and T-bet in immunopathogenesis of ankylosing spondylitis-novel targets for therapy? Front Immunol. (2019) 9:3132. doi: 10.3389/fimmu.2018.03132 30687330 PMC6335330

[78] Abdollahi E Tavasolian F Momtazi-Borojeni AA Samadi M Rafatpanah H. Protective role of R381Q (rs11209026) polymorphism in IL-23R gene in immune-mediated diseases: A comprehensive review. J Immunotoxicol. (2016) 13:286–300. doi: 10.3109/1547691X.2015.1115448 27043356

