# Supplementary figures and images for: Genetic associations in ankylosing spondylitis: circulating proteins as drug targets and biomarkers
Source: Front Immunol. 2024 May 21;15:1394438. doi: 10.3389/fimmu.2024.1394438 (PMC11148386; doi:10.3389/fimmu.2024.1394438)

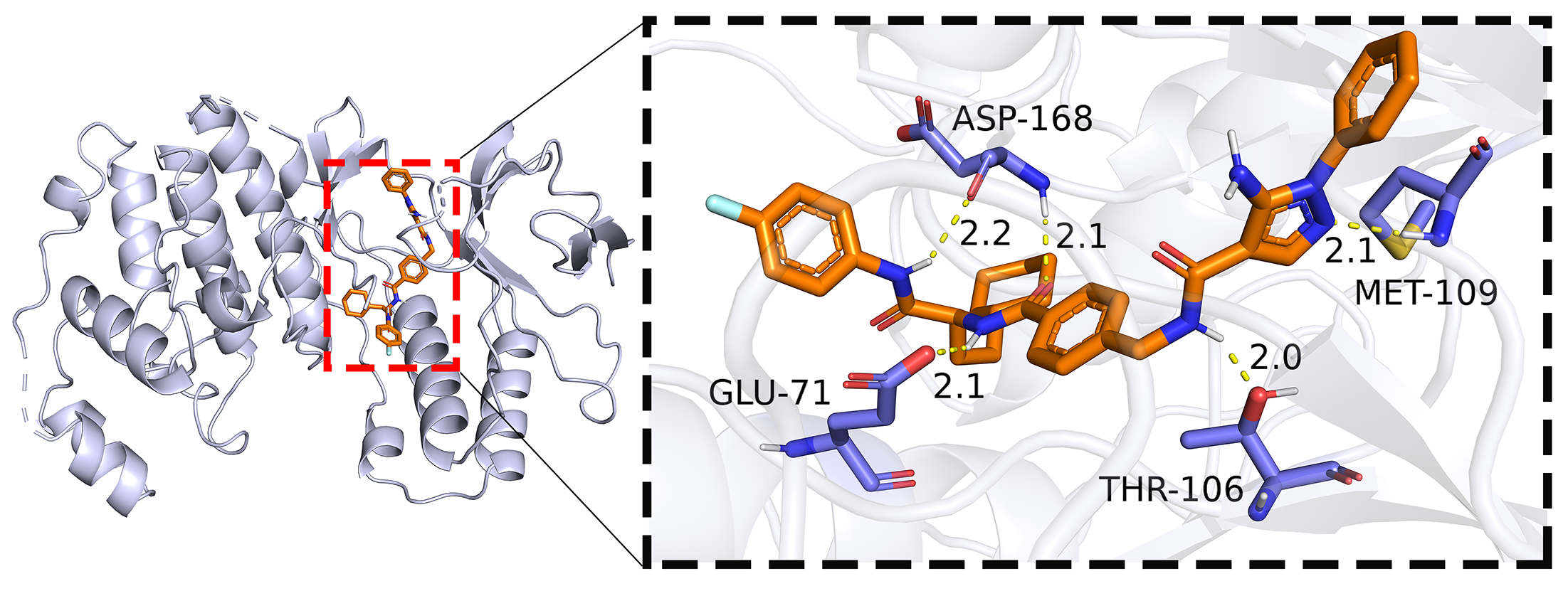

Supplement: Supplementary Figure 1 — Molecular docking Analysis of MAPK14 protein with a co-crystal ligand. The left panel shows the overall structure of the MAPK14 protein. The right panel illustrates the specific interactions between the co-crystal ligand and key amino acid residues in the MAPK14 protein. The panel represents the protein in a ribbon diagram, with the ligand depicted in a stick model, focusing on the binding site. The yellow dashed lines represent potential hydrogen bond contacts with key amino acids, with distances given in angstroms. quantitative trait loci (pQTLs), Instrumental variables (IVs), single nucleotide polymorphism (SNP), linkage disequilibrium (LD), mendelian randomization (MR), Wald ratio (WR), ankylosing spondylitis (AS), gene ontology (GO), Kyoto encyclopedia of genes and genomes (KEGG), Drug gene interaction database (DGIdb), Phenome-wide association study (PheWAS),Molecular docking (MD), major histocompatibility complex class I (MHC) chain-related protein A (MICA), mitogen-activated protein kinase 14 (MAPK14), activating transcription factor 6B (ATF6B), Allograft inflammatory factor 1 (AIF1), NK receptor group 2 member D (NKG2D), natural killer (NK), Apoptosis-associated speck-like protein containing a CARD (Act1), endoplasmic reticulum (ER), human leukocyte antigen B27 (HLA-B27), dendritic cells (DCs), regulatory T cells (Tregs), Macrophages (MØ), Interleukin 17 (IL-17), Interleukin 23 (IL-23), Interleukin 10 (IL-10), T-helper (Th) cells, Forkhead box protein P (FoxP), Antigen presenting cell (APC). [file Image_1.tif]
